# Supplementary material for: Exploring OR2H1-Mediated Sperm Chemotaxis: Development and Application of a Novel Microfluidic Device
Source: Cells. 2025 Jun 20;14(13):944. doi: 10.3390/cells14130944 (PMC12248556; doi:10.3390/cells14130944)
Supplement: Supplementary file 1 [file cells-14-00944-s001.zip › suppl figure S2.pdf]

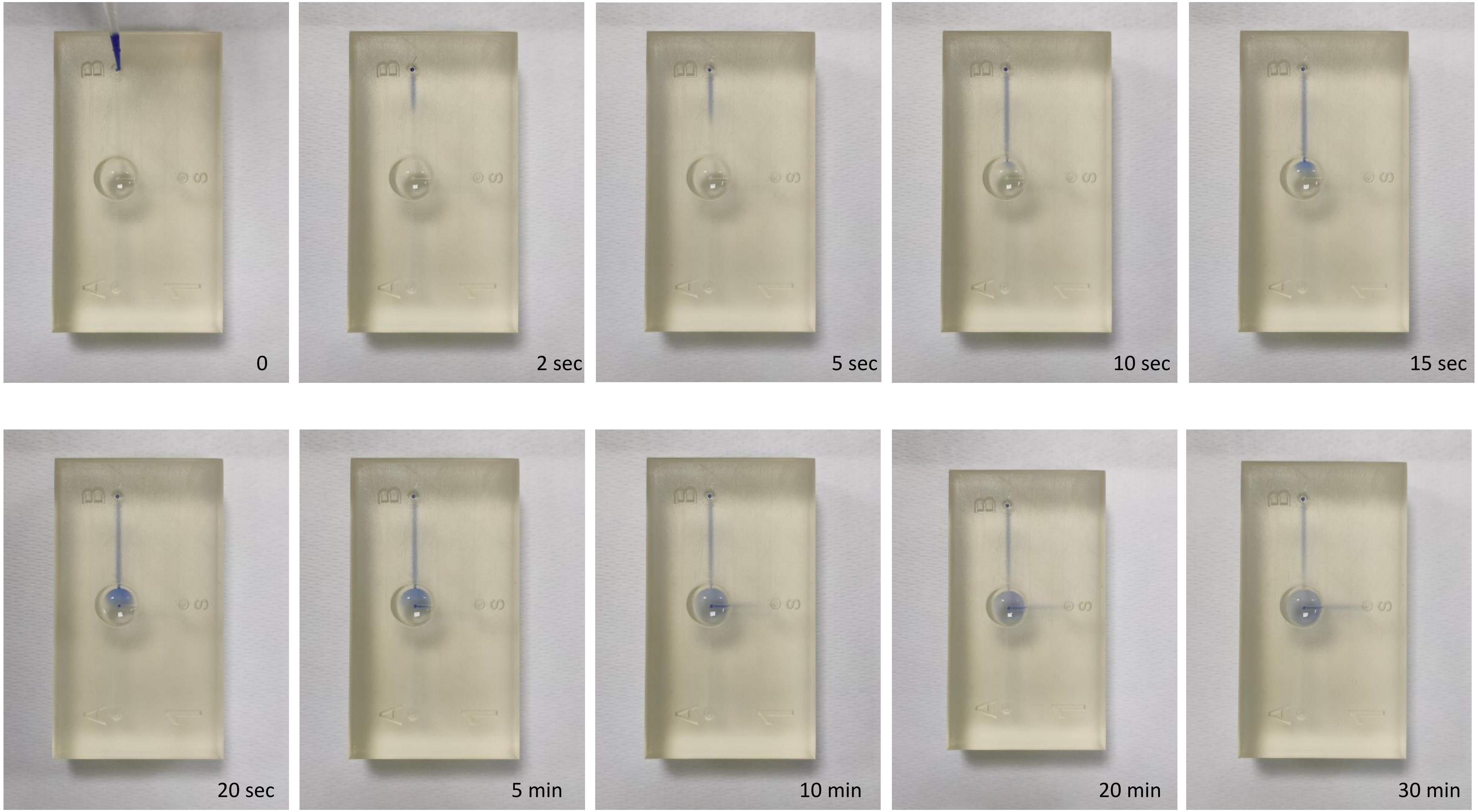

Supplementary figure S2. Evaluation of the time needed to form and maintain the chemical gradient. To this end, we visually estimated how long a loading dye (5x DNA loading dye, 5400200-VWR) needs to diffuse in the central well (20 seconds). Then we monitor the stability of the gradient: it remains stable throughout 30 minutes.
