# Supplementary material for: Exploring OR2H1-Mediated Sperm Chemotaxis: Development and Application of a Novel Microfluidic Device
Source: Cells. 2025 Jun 20;14(13):944. doi: 10.3390/cells14130944 (PMC12248556; doi:10.3390/cells14130944)
Supplement: Supplementary file 1 [file cells-14-00944-s001.zip › suppl figure S3.pdf]

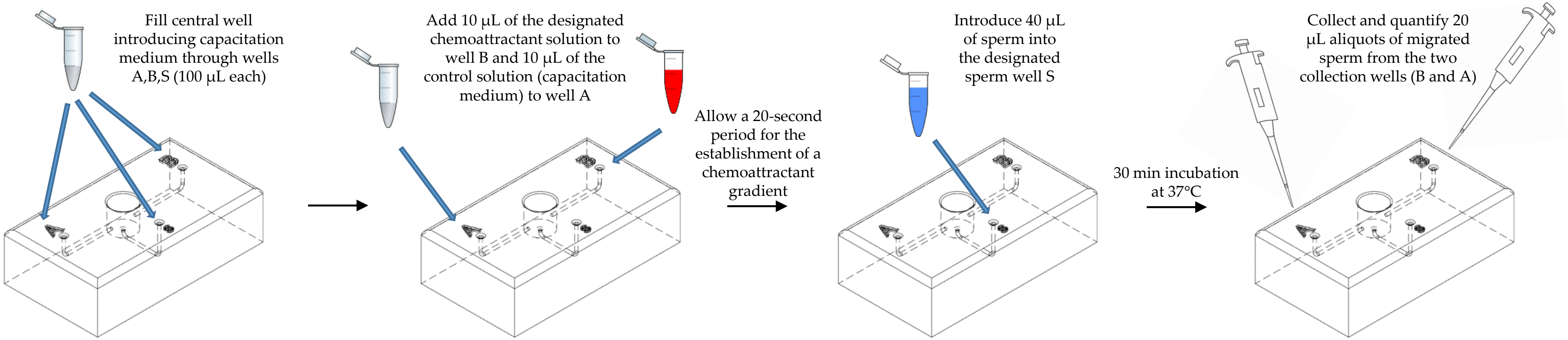

Supplementary figure S3. Protocol details: cartoons represent the steps of the working protocol (see main text).
