# Supplementary material for: Exploring OR2H1-Mediated Sperm Chemotaxis: Development and Application of a Novel Microfluidic Device
Source: Cells. 2025 Jun 20;14(13):944. doi: 10.3390/cells14130944 (PMC12248556; doi:10.3390/cells14130944)
Supplement: Supplementary file 1 [file cells-14-00944-s001.zip › suppl figure S4.pdf]

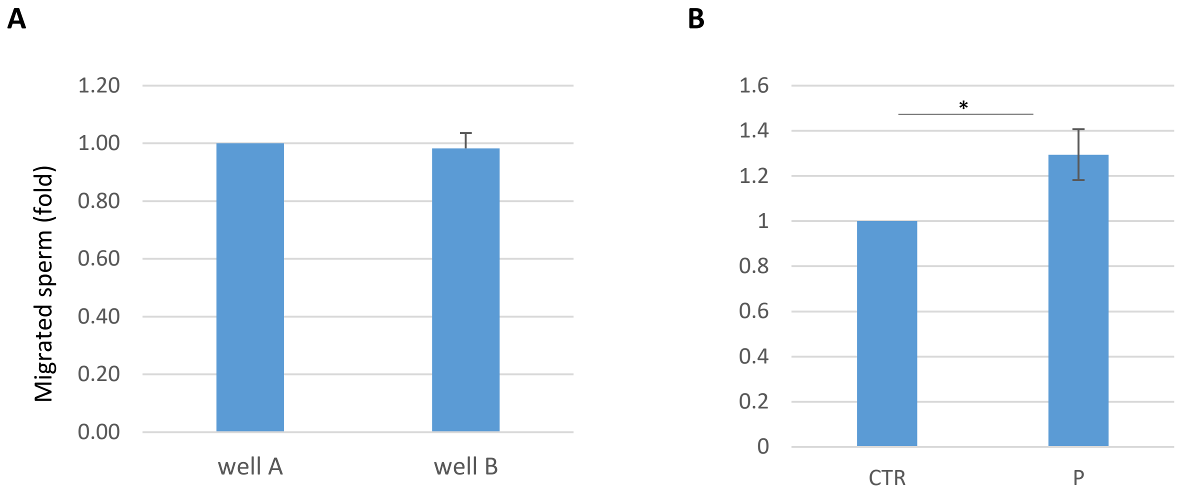

Supplementary figure S4. A. Histogram shows the migrated spermatozoa recovered in the well A and well B in the absence of chemoactrant. Data are presented as fold of recovered sperm relative to well A. The number of sperm cells recovered in well A is arbitrarily set to 1. Mean $\pm$ SD of three independent experiments are shown. **B.** Reverse-gradient, the chemoattractant is placed in the opposite well. Histogram shows the migrated spermatozoa recovered in the well A (P, Progesterone) and well B (CTR). Data are presented as fold of recovered sperm upon Progesterone treatment relative to CTR untreated well. The number of sperm cells (CTR) recovered in the untreated well is arbitrarily set to 1. Mean $\pm$ SD of three independent experiments are shown (N = 3, * = $p < 0.05$, one sample t-test).
