# Supplementary material for: Exploring OR2H1-Mediated Sperm Chemotaxis: Development and Application of a Novel Microfluidic Device
Source: Cells. 2025 Jun 20;14(13):944. doi: 10.3390/cells14130944 (PMC12248556; doi:10.3390/cells14130944)
Supplement: Supplementary file 1 [file cells-14-00944-s001.zip › suppl figure S5.pdf]

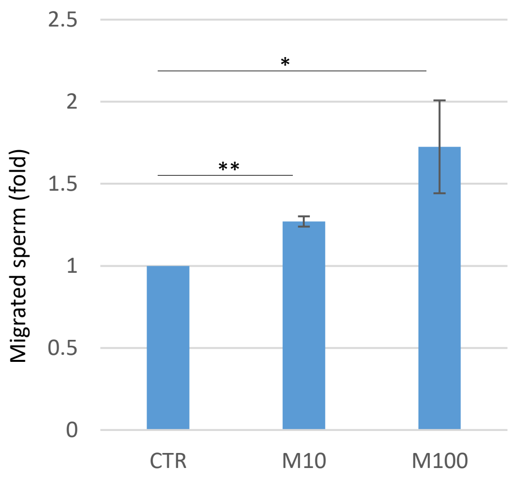

Supplementary figure S5. Histogram shows the migrated spermatozoa recovered in the well A (CTR) and well B (M10 or M100, Methional 10µM and 100µM). Data are presented as fold of recovered sperm upon different doses of Methional relative to CTR untreated well. The number of sperm cells (CTR) recovered in the untreated well is arbitrarily set to 1. Mean ± SD of five independent experiments are shown (N = 3, ** = p < 0.01, * = p < 0.05, one sample t-test).
