# Supplementary material for: Exploring OR2H1-Mediated Sperm Chemotaxis: Development and Application of a Novel Microfluidic Device
Source: Cells. 2025 Jun 20;14(13):944. doi: 10.3390/cells14130944 (PMC12248556; doi:10.3390/cells14130944)
Supplement: Supplementary file 1 [file cells-14-00944-s001.zip › Supplementary Figure S1.pdf]

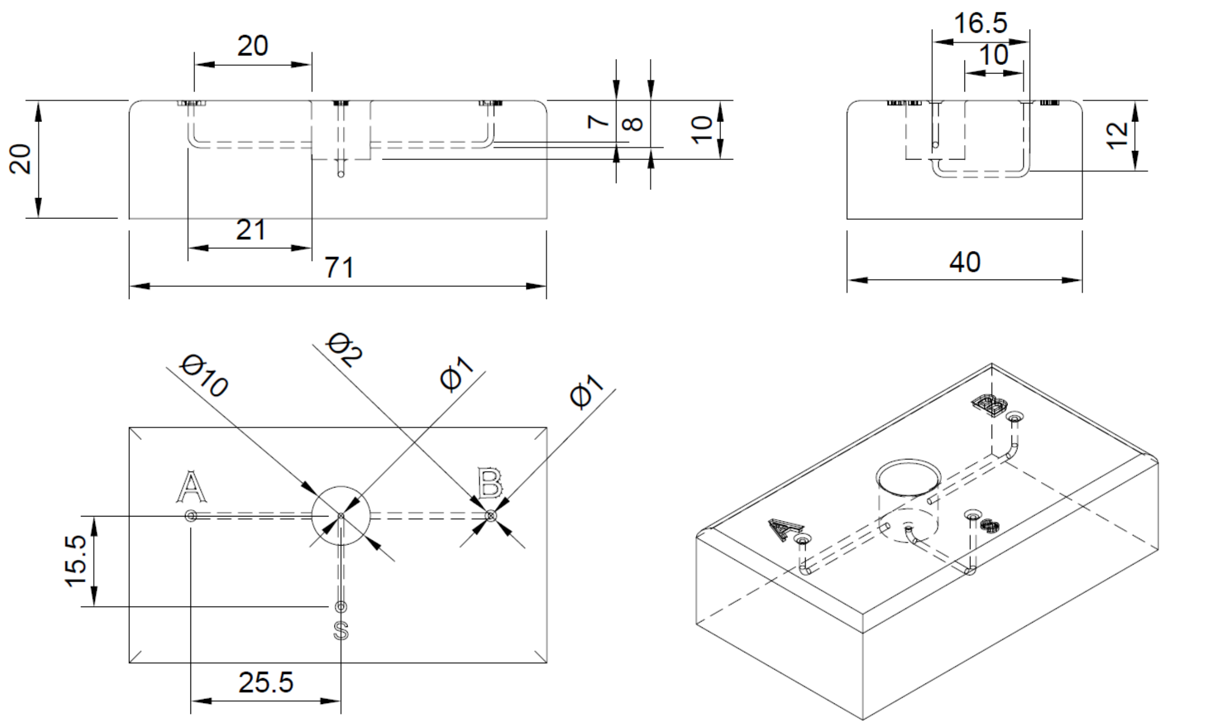

Supplementary Figure S1. Detailed design of the sperm chamber. Dimensions are reported in millimeters. All channels have the same diameter (1 mm). Channels A and B are identical in length.
