# Supplementary material for: Exploring OR2H1-Mediated Sperm Chemotaxis: Development and Application of a Novel Microfluidic Device
Source: Cells. 2025 Jun 20;14(13):944. doi: 10.3390/cells14130944 (PMC12248556; doi:10.3390/cells14130944)
Supplement: Supplementary file 1 [file cells-14-00944-s001.zip › Supplemetary file S1A 3D Printing files.pdf]

SpermChamber.stl: a ready to print .stl file of a sperm chamber as used in the experiment. The project was developed using Autodesk® Fusion360 (San Rafael, CA, USA). We suggest to 3D print the device with high resolution (e.g. 29-micron accuracy) in a biocompatible resin (e.g. VisiJet® Crystal, EX 200 Plastic Material, USP Class VI certified for medical applications; 3D Systems, Rock Hill, South Carolina, USA) using the lost-wax method. The file can be inspected with any 3d development software (Microsoft 3D Viewer, Apple Preview, Paint 3D and many others).
